# Anemia in Children with Down Syndrome

**DOI:** 10.1155/2011/813541

**Published:** 2011-09-14

**Authors:** Ariel Tenenbaum, Sarah Malkiel, Isaiah D. Wexler, Floris Levy-Khademi, Shoshana Revel-Vilk, Polina Stepensky

**Affiliations:** ^1^Department of Pediatrics, Hadassah University Medical Center, Mount Scopus Campus, Jerusalem, Israel; ^2^Department of Pediatric Hematology Oncology, Hadassah University Medical Center, Ein Kerem Campus, Jerusalem, Israel

## Abstract

*Background*. Iron deficiency anemia impacts on cognitive development. The objective of this study was to determine the prevalence of anemia and iron deficiency in children with Down syndrome and identify risk factors for anemia. *Methods*. We conducted a prolective cross-sectional study of children attending a multidisciplinary Down syndrome medical center. One hundred and forty nine children with Down syndrome aged 0–20 years were enrolled in the study. Information obtained included a medical history, physical and developmental examination, nutritional assessment, and the results of blood tests. *Results*. Of the patients studied, 8.1% were found to have anemia. Among the 38 children who had iron studies, 50.0% had iron deficiency. In a multivariate analysis, Arab ethnicity and low weight for age were significantly associated with anemia. Gender, height, the presence of an eating disorder, and congenital heart disease were not risk factors for anemia. *Conclusions*. Children with Down syndrome are at risk for anemia and iron deficiency similar to the general population. Children with Down syndrome should be monitored for anemia and iron deficiency so that prompt intervention can be initiated.

## 1. Introduction

Iron deficiency anemia (IDA) is prevalent and a major public health care issue [1, 2]. IDA has been associated with motor and cognitive developmental deficits in children and may be irreversible [3–7]. IDA may have an even greater impact on select populations who are already at risk for intellectual and developmental compromise.

Down syndrome (DS) is the most common human aneuploidy with an approximate incidence of one in 800 live births. Clinical manifestations of DS include cognitive impairment, craniofacial dysmorphism, gastrointestinal tract abnormalities, congenital heart defects, endocrine abnormalities, immunologic defects, and neurologic deficits associated with early onset dementia [8]. Regarding the hematopoietic system, children with DS frequently show macrocytosis, abnormalities in platelet counts, and an increased prevalence of leukemia [9–13]. Similar to the general population, individuals with DS may be at risk for IDA and its consequent complications.

There are only a few studies regarding anemia in DS patients. Until recently, those studies which have been published often focused on a select population of children with DS and may not be representative of the general DS population. For example, Awasthi et al. investigated the hematological profile of patients with DS who presented with initial manifestations of a hematological disorder to a genetics clinic [14]. Of the 239 children with DS who were examined over a 10-year period, 6.2% were found to have a hematological disorder. Of those 6.2%, 73.3% had acute leukemia or transient myeloproliferative disorder (TMD). Only 4 of the 239 children in the study (1.7%) were found to have anemia. Henry et al. performed blood counts on 159 newborns (aged 0–7 days) with DS. These tests revealed the prevalence of anemia to be no different from the general neonatal population. The authors suggested that anemia in this age group may be more of a reflection of either maternal iron stores or delivery-related newborn blood losses [15].

Dixon et al. performed blood counts and iron studies on 114 children with DS and found IDA in 3% and ID in 10%, comparable with their prevalence in the general pediatric population [16]. 

The paucity of data on the prevalence of anemia in DS patients of different age groups and the deleterious consequences associated with IDA lead us to investigate the prevalence of anemia in this population and the specific risk factors associated with IDA in children with DS.

## 2. Methods

### 2.1. Participants

During 2004–2007, 149 children and young adults with DS were seen at the Center for Down Syndrome at the Hadassah Mount Scopus University Medical Center, and these individuals were included in the study. The Center for Down Syndrome serves the entire population of Israel, and patients from the predominantly Arab neighborhoods of East Jerusalem are actively recruited as part of a cooperative (Israeli/Palestinian physicians) community-based program to enhance the care of children with DS from all ethnic backgrounds. As such, the center's patient population is representative of the general population of the region. The study protocol was approved by the Hadassah-Hebrew University Institutional Review Board (Helsinki Committee).

The study was prolective, with the patient population and data parameters defined before data acquisition. The patient population included children and young adults with DS aged 0–20 years at their first visit to the Center (before any intervention). Patients were eligible for the study if they had a complete blood count (CBC) performed as part of their evaluation or had undergone blood testing within 3 months of their visit (as part of the center's protocol, we request that parents or patients bring recent laboratory results). It should be noted that all laboratory studies for outpatients in Israel (there is universal health coverage for Israeli citizens) must be approved by the patient's health maintenance organization (HMO), and HMOs have different policies regarding approval of laboratory testing. A subgroup of 38 children also had iron studies (iron, ferritin, and transferrin levels) either performed at our center or at the child's HMO. All participants arrived at the center for a general medical evaluation.

### 2.2. Measures

A complete medical history was taken and patients underwent a physical examination. Information collected for the study included birth history, initial nutrition, motor development, congenital malformations, and DS-related complications (e.g., feeding disorders, oropharyngeal hypotonia, etc.). Anthropomorphic data was recorded including BMI. Height and weight measurements were analyzed in the context of growth charts developed for individuals with DS. The nutritional status of the patients was assessed, and every patient was evaluated for the presence of a feeding or nutritional disorder by the center's dietitian and speech therapist.

### 2.3. Statistical Methods

All hematinic study data was analyzed in terms of established age appropriate norms based on the NHANES III [1]. Data was entered into a spreadsheet and imported to the SPSS statistical program (SPSS 15, Chicago). Both nonparametric and parametric statistical tools were used to analyze the data as appropriate. Associations between categorical variables were explored using the *χ* ^2^ test or Fisher's exact test. Categorical variables found to be associated with anemia in univariate analysis were further analyzed in a logistic regression model.

## 3. Results

During the years 2004–2007, 303 children and young adults were evaluated as first-time patients at the Center for Down Syndrome. Of these, 149 (49.0%) had the requisite data to be included in the study. The demographic and clinical data of the study group is shown in Table 1. There was a greater proportion of males which is to be expected, as there is male predominance among patients with DS. The division between Jewish and Arab patients was close to the population distribution in the region. More than half the patients had heart defects, feeding disorders, and motor delay. With regard to feeding disorders and delayed walking, not all parents could recall this information or children had not reached an age at which this could be determined.

The overall prevalence of anemia in our study group was 8.1% (Table 2). The proportion of children with anemia increased with age, with 13.7% of children over the age of 10 years having anemia. Likewise, the number of children having blood hemoglobin that was lower than the published NHANES III averages for different age groups increased with age.

Hematologic indices for the study group are shown in Table 3. The majority of children did not have anemia or microcytosis. Iron stores as measured by serum ferritin indicated that nearly all children (26/27) tested had below normal levels for age. In contrast, 26 children had their serum transferrin measured, and their levels were normal for their age.

### 3.1. Risk Factors for Anemia

In univariate analysis, it was found that age, ethnicity, weight, and motor development were related to an increased risk for anemia. Height, history of feeding disorder, or congenital heart defects did not increase the risk for anemia.

To determine the adjusted odds ratio for anemia, a logistic regression model was utilized (Table 4). The most significant risk factor for anemia was ethnicity. Patients of Arabic ethnicity were found to have a nearly 5-fold increase in anemia. Weight also had an impact on anemia, with the rate of anemia decreasing by 0.03 with every increase by one percentile in weight.

We also investigated variables associated with having a below average hemoglobin for age. Interestingly, an association between motor development and below average hemoglobin concentration for age was found. When looking at the age of independent walking in 53 children, a lower than average hemoglobin for age was more prevalent among those who started walking after the age of 2 years (53.1% versus 24.0%, *P* < 0.05).

## 4. Discussion

IDA is relatively common in the general population, and in many countries and states, there are screening programs to identify children with anemia. The reason for the heightened concern regarding anemia is that IDA has been associated with motor and cognitive developmental deficits in children [3–7]. In children with DS, whose intellectual capacity is compromised to begin with, utmost attention should be given to preventing anemia and its potentially deleterious developmental consequences.

In the present study, we show that the prevalence of anemia in a general population of children with DS is 8.1%, with the rate increasing with age. Factors associated with anemia in this study included ethnicity and low weight.

There is a question of whether the relatively high rate of anemia in our population is unique to DS or more a reflection of the general population. Review of studies done in the general Israeli population shows that IDA is an endemic problem (similar to other countries in the Middle East) [17–24], and it appears that the rates of anemia for patients with DS in our clinic population were lower than those of the general population. For example, Meyerovitch et al. found anemia to be as high as 15.5% in 9–18-months old infants [22]. Fraser et al. found a similar prevalence of 15% among Jewish children and approximately 25% among Bedouin children [23]. Bilenko et al. reported a prevalence as high as 47% [24]. It should be noted that the great variability between studies may represent regional differences and different criteria for defining anemia.

The higher prevalence of anemia among Arab children with DS reflects a more wide-ranging trend among the general population. Lavon et al. found a higher prevalence in Arab children: among Jewish children, the rate of anemia was 44.3% from birth to 6 months and increased to 60% from the age of 6 months to one year. For similar age groups in the Arab population, the rates were 43.7% and 71% [25]. Similar differences between groups have been noted in other studies [18, 23]. Meyerovitch et al. found that both Arab and ultra-Orthodox Jews had increased rates of IDA [22]. It is possible that ethnic and religious differences related to anemia are secondary to socioeconomic factors which have an impact on adherence to Health Ministry recommendations regarding iron supplementation [26].

The association that we found between low weight and anemia supports the hypothesis that inadequate nutrition may contribute to IDA and even explain some of the differences found between different ethnic populations. Cause and effect are difficult to determine, as severe iron deficiency alone may cause anorexia and poor weight gain. Other factors, such as various chronic diseases which are more prevalent in DS may also contribute to anemia. As part of our initial evaluation of the participants, we ruled out celiac disease, thus excluding this condition as a contributing factor to IDA. However, it is possible that feeding disorders, which are common among children with DS, may have caused nutritional deficits leading to anemia.

The prevalence of anemia is declining among the general pediatric population in Israel [21, 22]. Public health initiatives have played a role in lowering the rate of anemia, and children with DS have most likely benefited from these interventions which include iron supplementation for all children aged 4 months to one year, with continuation of supplementation if the CBC at one year of age indicates anemia.

There are several limitations to the present study. The results of iron studies were available for only 38 of the 149 children we evaluated for anemia in this study. Thus, we cannot truly characterize the anemia we found as IDA, or identify risk factors associated with iron deficiency. We are further limited in this by the presence of relatively high MCV in this population, as discussed further below. Thalassemia trait also remains a possible etiology for anemia in this ethnic population. However, as iron deficiency is the most common cause of anemia in all age groups worldwide, and in the absence of other proven causes of anemia in our study population, iron deficiency remains the most likely cause of the anemia found in this study. 

The study population consisted of children who are in the care of the Center for Children with Down Syndrome. It is possible that they do not adequately represent the general population of children with DS in Israel. Our population may overrepresent families with high compliance for medical followup and treatment, and for this reason, they may have a lower prevalence of anemia than other children with Down syndrome. On the other hand, children with relatively mild manifestations of DS may be underrepresented in our clinic so that our study population is biased towards children with more severe manifestations of DS which may impact the rates of anemia.

An interesting finding in our study was the tendency to macrocytosis in all age groups. Macrocytosis is a common finding in children with DS and has been widely reported [16, 27–29]. Macrocytosis is not related to Vitamin B12 or folic acid deficiencies, and most likely, there is a metabolic or genetic reason for the macrocytosis. As a result, there may be difficulty in completely characterizing IDA due to macrocytosis. The clinical significance of this point is that the iron status of children with DS should be monitored by both serial CBCs and iron studies.

## 5. Conclusions

The prevalence of IDA in children with DS does not greatly differ from the general population. Nevertheless, children with DS and IDA might be more susceptible to the effects of anemia based on the fact that they already have DS-related deficits in motor and neurocognitive development. Since IDA is an easily preventable and treatable problem when diagnosed in a timely manner, it is important to monitor children with DS by periodically performing a CBC and measuring iron stores.

## Figures and Tables

**Table 1:** Demographic and clinical characteristics of patients.

Gender (M : F)	1.78
Age (mean, S.D., & range)	7.13 ±.53 y
Age distribution (*n*, %)	0–20 y
0–2	41 (27.5)
2–6	42 (28.2)
6–10	15 (10.1)
≥10	51 (34.2)

Ethnicity	
Jewish	127 (85.3)
Arabic	22 (14.7)

Heart defects	80/149 (53.7)

Feeding disorders	23/41 (56.1)

Motor delay	32/57 (56.1)

**Table 2:** Blood counts and iron studies (by age group).

	Hemoglobin	MCV	Iron	Ferritin	Transferrin
**0–2**					
Mean ± SD	13.38 ± 2.93	86.32 ± 8.76	36.13 ± 17.65	31.70 ± 21.77	254.80 ± 63.05
Range	8.80–22.50	73.10–110.0	5.41–57.20	5.30–70.30	167.00–365.99
N	41	40	9	9	7

**2–6**					
Mean ± SD	12.55 ± 1.13	84.44 ± 6.54	62.03 ± 32.68	19.47 ± 9.96	309.88 ± 36.80
Range	9.30–15.10	58.80–94.90	23.00–112.68	7.58–33.00	266.50–360.25
N	42	41	10	7	6

**6–10**					
Mean ± SD	13.30 ± 1	87.53 ± 4.73	60.40 ± 20.64	29.27 ± 18.24	270.81 ± 51.32
Range	11.0–14.80	75.80–94.10	28.70–89.00	4.28–46.10	207.00–377.72
N	15	15	6	4	5

**≥10**					
Mean ± SD	13.91 ± 1.4	90.17 ± 5.48	75.60 ± 63.66	53.09 ± 59.02	271.43 ± 34.54
Range	9.80–16.40	68.90–102.70	27.30–279.00	4.90–170.00	224.34–330.46
N	51	49	13	7	8

**Table 3:** Anemia and low hemoglobin for age in age groups.

Age (years)	Anemia *N* (%)	Low hemoglobin for age*
0–2	2/41 (4.9%)	13/41 (31.7%)
2–6	2/42 (4.8%)	12/42 (28.6%)
6–10	1/15 (6.7%)	6/15 (40%)
>10	7/51 (13.7%)	33/51 (64.7%)

Total	12/149 (8.1%)	64/149 (43%)

*Lower than the published NHANES III age-related averages for hemoglobin.

**Table 4:** Adjusted odds ratio (OR) for anemia in children with DS.

	B	S.E.	Sig.	Adjusted OR (C.I. 95%)
Ethnicity	−1.483	0.709	0.036	0.227 (.057–.911)
Age	0.145	0.051	0.004	1.156 (1.047–1.277)
Weight	−0.032	0.013	0.015	0.968 (0.944–0.994)
Constant	−1.214	0.735	0.099	0.297
